# Supplementary material for: Different populations of CD11b+ dendritic cells drive Th2 responses in the small intestine and colon
Source: Nat Commun. 2017 Jun 9;8:15820. doi: 10.1038/ncomms15820 (PMC5472728; doi:10.1038/ncomms15820)
Supplement: Supplementary Information [file ncomms15820-s1.pdf]

Type of file: PDF
Size of file: 0 KB
Title of file for HTML: Supplementary Information
Description: Supplementary figures

Type of file: pdf
File size:
Title of file for HTML: Peer Review File
Description:

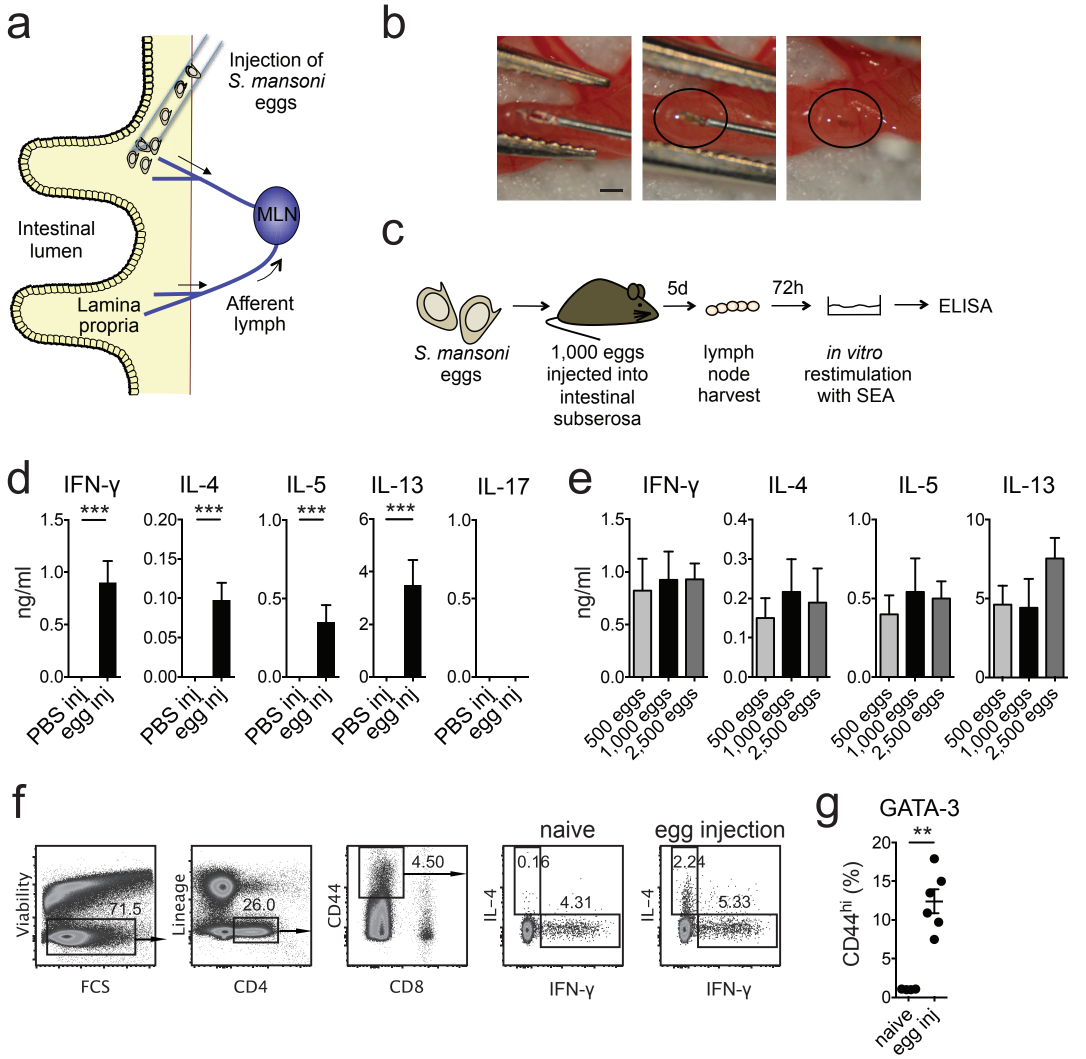

Supplementary Figure 1. Intestinal Th2 responses to *Schistosoma mansoni* eggs induce antigen specific IFN-γ and Th2 responses. (**a**) Schematic illustrating the injection of *S. mansoni* eggs into the intestine. (**b**) Photographs taken during the injection procedure into the murine small intestine. Insertion of the syringe into the subserosal layer (left panel), injection of the eggs (middle panel) and deposited eggs after the removal of the syringe (right panel) are shown. Scale bar corresponds to 1 mm. (**c**) Schematic illustrating the experimental set up for immunization. 1,000 *S. mansoni* eggs were injected into the subserosal layer of the intestine and resulting T cell responses were analyzed after 5 days by restimulating mesenteric lymph node (MLN) cells for an additional 3 days in the presence of schistosome egg antigen (SEA) and measuring the released cytokines by ELISA. (**d**) Cytokine responses of restimulated MLN cells from PBS injected (PBS inj) and egg injected (egg inj) mice (n=9 mice per group, in three independent experiments, mean ± SEM, Mann-Whitney *U* tests, ***P≤0.001). (**e**) Cytokine responses of restimulated MLN cells from egg injected mice, where 500, 1,000 or 2,500 eggs were injected (n=4-6 mice per group, in two

independent experiments, mean ± SEM, Kruskal Wallis tests (not significant)). (**f**) Corresponds to data shown in Fig. 1b. Representative gating strategy of MLN cells harvested from egg injected animals five days after immunization. CD44^{hi} CD4 T cells were identified by flow cytometry and levels of IFN-γ, IL-4 and IL-13 cytokine production measured after PMA/ionomycin stimulation and compared to cells harvested from naive animals. (**g**) Five days after immunization with *S. mansoni* eggs (egg inj), MLNs were harvested, CD44^{hi} CD4 T cells identified as in (**f**) and levels of the Th2 associated transcription factor GATA-3 measured by flow cytometry and compared to cells harvested from naive animals (n=6 mice per group, in two independent experiments, mean ± SEM, Mann-Whitney *U* tests, **P≤0.01).

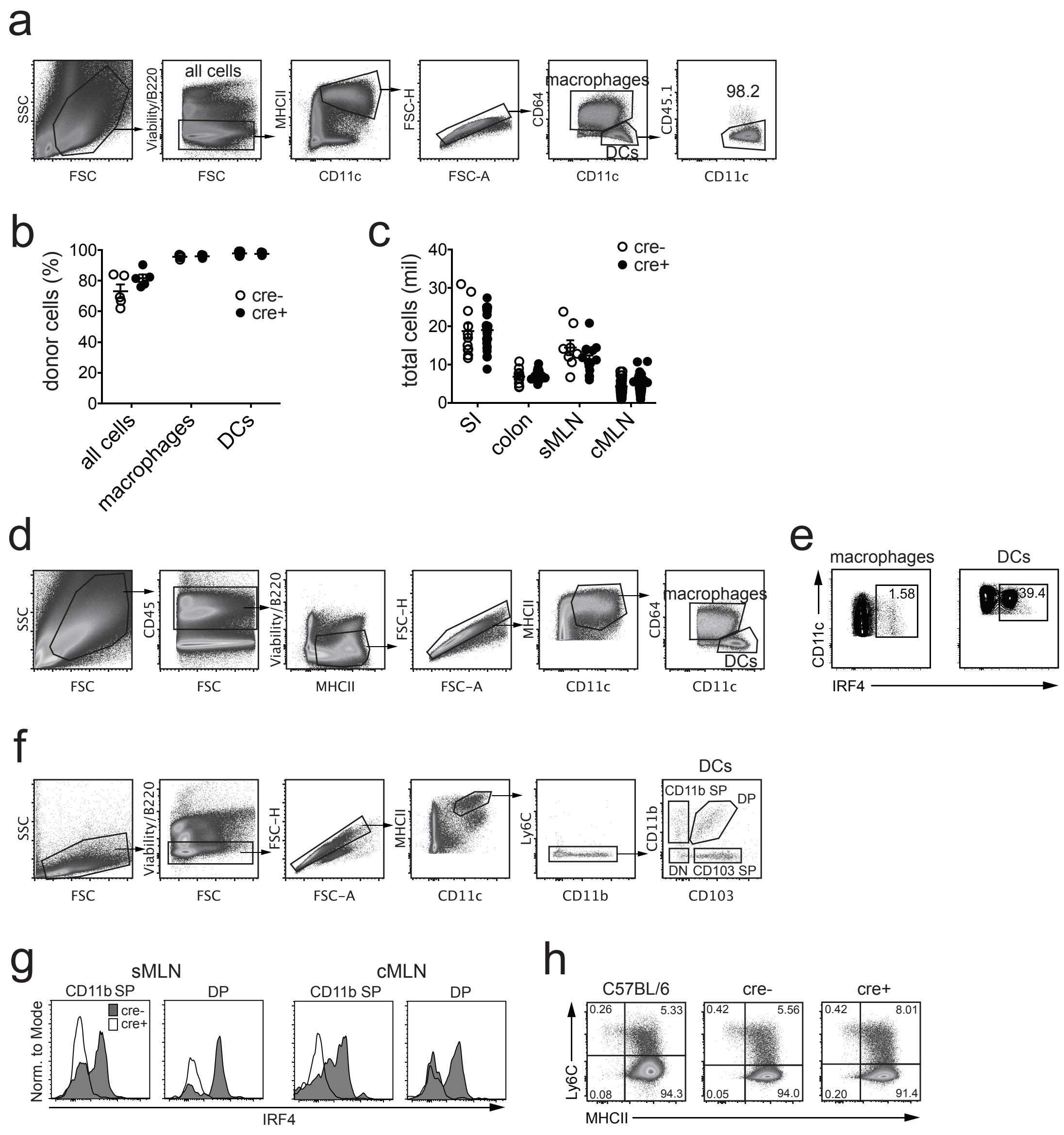

Supplementary Figure 2. Intestinal DCs and macrophages are completely reconstituted in IRF4$^{f/f}$ CD11c-cre positive and cre-negative bone marrow chimeric mice. (**a**) Representative gating strategy identifying small intestinal macrophages and dendritic cells (DC) from IRF4$^{f/f}$ cre-negative (cre-) bone marrow (BM) chimeric mice. (**b**) The percentage of reconstitution was measured in IRF4$^{f/f}$ CD11c-cre positive (cre+) or littermate cre- BM chimeric mice. Live cells, intestinal macrophages and DCs, as gated in (**a**), were analyzed (n=5 mice per group, in two independent experiments, mean ± SEM, Mann-Whitney *U* tests (not significant)). (**c**) Total cell numbers of enzymatically digested small intestines, colons and their respective draining lymph nodes were compared between cre+ or littermate cre- BM chimeras (n=8-20 mice per group, in more than three independent experiments, mean ± SEM, Mann-Whitney *U* tests (not significant)). (**d**) Corresponds to data shown in Fig. 2d. Representative gating strategy identifying small intestinal macrophages and DCs from C57BL/6 mice. (**e**) IRF4 expression by intestinal macrophages and DCs, as gated in (**d**), analyzed by flow cytometry in

C57BL/6 mice, and gated on the appropriate isotype control. (**f**) Representative gating strategy of enzymatically digested small intestinal mesenteric lymph node (sMLN) cells of cre- animals to identify migratory DCs and their CD11b and CD103 expressing populations. (**g**) Representative IRF4 expression of sMLN and colonic MLN (cMLN) $CD11b^{+}CD103^{-}$ single positive (CD11b SP) and $CD11b^{+}CD103^{+}$ double positive (DP) DCs from cre+ and cre- animals, as gated in (**f**). (**h**) Representative FACS plots depicting $Ly6C^{hi}$ monocytes and mature $Ly6C^{-}$ $MHCII^{+}$ macrophages in C57BL/6 mice and cre+ and cre- BM chimeras.

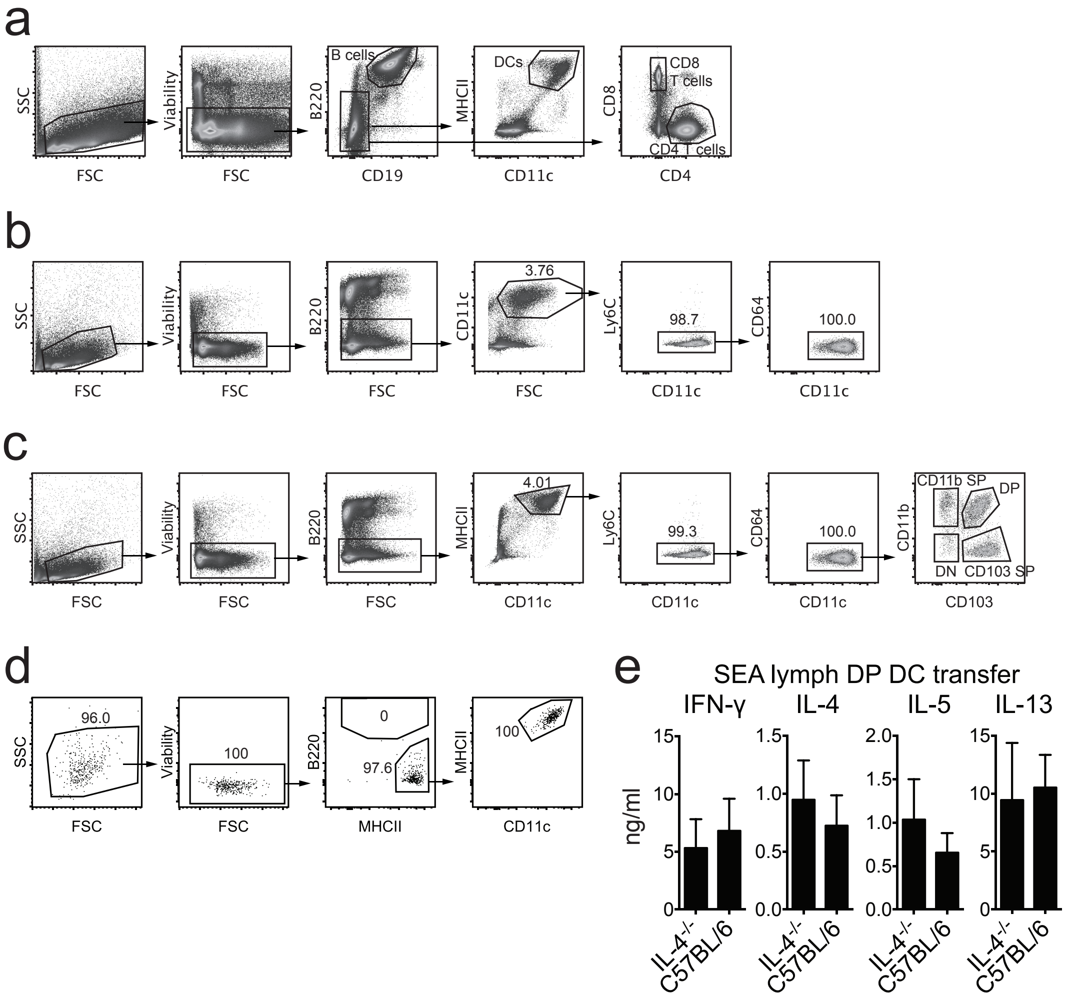

Supplementary Figure 3. Lymph migrating DCs can be identified in intestinal draining lymph and can induce Th2 responses independent of their expression of IL-4. (**a**) Corresponds to data shown in Fig. 2a,b. Representative gating strategy of lymph migrating CD4 and CD8 T cells, B cells and dendritic cells (DCs) in intestinal draining lymph collected from mesenteric lymphadenectomized (MLNx) C57BL/6 animals for 18 hours. (**b**) Corresponds to data shown in Fig. 2c. Representative gating strategy of lymph migrating CD11c^{hi} CD64^{-} B220^{-} DCs from MHCII$^{-/-}$ MLNx mice. (**c**) Corresponds to data shown in Fig. 2d and 3a. Representative gating strategy of lymph migrating MHCII^{hi} CD64^{-} B220^{-} CD11c^{hi} DCs from MLNx mice, identifying the four CD11b and CD103 expressing DC populations. (**d**) Representative sort purity of lymph migrating MHCII^{hi} B220^{-} CD11c^{hi} DCs from MLNx mice. (**e**) 30,000 DP DCs from the lymph of C57BL/6 or IL-4$^{-/-}$ MLNx mice were incubated with schistosome egg antigen (SEA) for 18 hours *in vitro* and transferred under the mesenteric lymph node (MLN) capsule of wild type recipient animals. Antigen specific T cell responses in the injected MLNs were measured 5 days after cell transfer by cytokine analysis of *in vitro* SEA restimulation cultures (n=6 mice per group, in three independent experiments, mean ± SEM, Mann-Whitney *U* tests (not significant)).

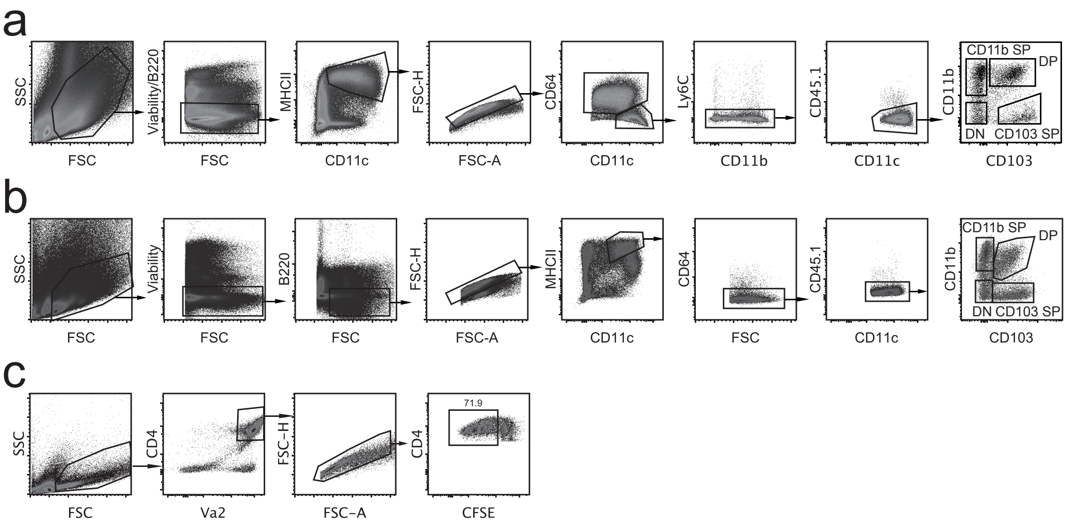

Supplementary Figure 4. CD11b and CD103 expressing DC populations can be identified in the intestine and lymph nodes of IRF4$^{f/f}$ CD11c-cre positive and cre-negative bone marrow chimeric mice. (**a**) Corresponds to data shown in Fig. 4a-d. Representative gating strategy for enzymatically digested small intestinal lamina propria cells of IRF4$^{f/f}$ cre-negative (cre-) bone marrow (BM) chimeric mice to identify CD11b and CD103 expressing dendritic cell (DC) populations. Equivalent analysis was performed in IRF4$^{f/f}$ CD11c-cre positive (cre+) animals and the colon. (**b**) Corresponds to data shown in Fig. 4e,f,h. Representative gating strategy of enzymatically digested small intestinal mesenteric lymph node (sMLN) cells of cre- chimeric mice to identify migratory DC populations. Equivalent analysis was performed in cre+ animals and in the colon draining colonic MLNs. (**c**) Corresponds to data shown in Fig. 4g. Representative gating strategy identifying proliferated OT-II CD4 T cells, by their diluted concentration of CFSE, after 3 days of coculture with OVA-pulsed cre+ or cre- MLN DC populations.

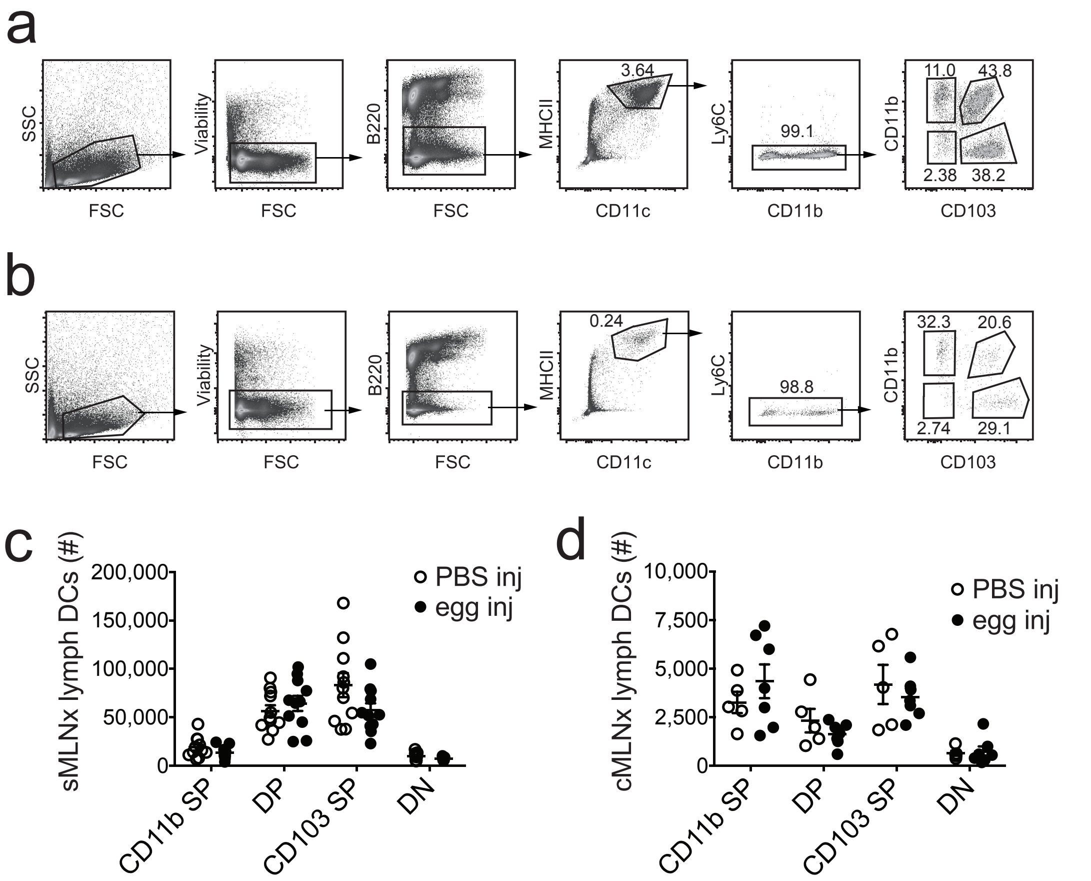

Supplementary Figure 5. CD11b and CD103 expressing dendritic cell populations can be identified in small intestinal and colonic draining lymph. (**a**) Corresponds to data shown in Fig. 5a-c. Representative gating strategy of lymph migrating dendritic cell (DC) populations from small intestinal mesenteric lymphadenectomized (sMLNx) C57BL/6 animals that specifically drain from the small intestine. (**b**) Corresponds to data shown in Fig. 5e,f. Representative gating strategy of colon draining lymph migrating DC populations from colonic mesenteric lymphadenectomized (cMLNx) mice. (**c**) Total number of lymph migrating DC populations, as gated in (**a**), 18 hours after the injection of PBS (PBS inj) or *Schistosoma mansoni* eggs (egg inj) into the small intestine of sMLNx animals (n=10-11 mice per group, in three independent experiments, mean ± SEM, Mann-Whitney *U* tests (not significant)). (**d**) Total number of lymph migrating DC populations, as gated in (**b**), 18 hours after the injection of PBS or *S. mansoni* eggs into the colon of cMLNx animals (n=6-7 mice per group, in three independent experiments, mean ± SEM, Mann-Whitney *U* tests (not significant)).
